# Addition of metabolomics to the omics spectrum for cancer biomarker development– demonstration with the KrasG12D mouse model for pancreatic carcinoma

**DOI:** 10.1186/1471-2164-15-S2-O4

**Published:** 2014-04-02

**Authors:** Evagelia C Laiakis, Joseph J LaConti, Amrita Cheema, Anton Wellstein, Albert J Fornace

**Affiliations:** 1 Department of Biochemistry and Molecular & Cellular Biology, Georgetown University Medical Center, Washington, DC 20057, USA; 2 Department of Oncology, Georgetown University Medical Center, Washington, DC 20057, USA

## 

Advancements in 'omics' technologies offer opportunities to complement current approaches in cancer diagnosis and assessment of response to therapy. While the relatively mature fields of transcriptomics and proteomics have demonstrated their utility in cancer diagnosis and prognosis, they typically rely on complicated and demanding methodologies requiring isolation of intact ribonucleic acid or protein and then analyses. Metabolomics is an emerging field with great potential for cancer screening and early diagnosis. Here metabolites, defined as small molecules typically less than 1 kDa, are assessed with modern analytical approaches that can detect thousands of different molecules, and identify informative subsets of manageable size for use in simpler cost-effective instrumentation in the field. Cancer cells show substantial perturbations in metabolism such as glycolysis, Warburg effect, and many laboratories are now assessing its utility in oncology.

In the case of pancreatic duct adenocarcinoma (PDAC), this tumor type is the fourth leading cancer worldwide with less than 6% 5-year survival rate. PDAC is associated with poor prognosis based on the late stage diagnosis of the disease, hence being referred to as the “silent killer”. Current diagnostic tests lack the sensitivity and specificity to identify markers of early staging, and efforts have been carried out at Georgetown University to identify metabolomic biomarkers in patient PDAC tissue which then can be assessed in biofluids such as urine and serum. To assess the feasibility of biomarker development in PDAC, we have also taken a mouse models approach using the p48-Cre/LSL-KrasG12D mouse model. This model shows malignant progression to PDAC analogous to the human disease stages via early and late pancreatic intra-epithelial neoplasia (PanIN) lesions. Serum was collected from mice with early PanIN lesions (at 3-5 months) and with late PanIN or invasive PDAC lesions (13-16 months), as determined by histopathology. Metabolomics analysis of the serum samples was conducted through Ultra Performance Liquid Chromatography coupled to Time-of-flight Mass Spectrometry (QTOF). Multivariate data analysis revealed distinct metabolic profiles between the pancreatic cancer progression stages. One of the metabolites was validated through tandem mass spectrometry and showed increased circulating levels in mice with late PanIN and PDAC. Immunohistochemical analysis of pancreatic tissue showed increased levels of the enzyme, which produces this metabolite, at the late stages of pancreatic cancer, correlating with circulating metabolite levels. The use of this approach in developing diagnostic strategies with metabolomics in the detection of at-risk subjects will be discussed as a complement to genomics approaches.

